# A Noninvasive TDR Sensor to Measure the Moisture Content of Rigid Porous Materials

**DOI:** 10.3390/s18113935

**Published:** 2018-11-14

**Authors:** Zbigniew Suchorab, Marcin Konrad Widomski, Grzegorz Łagód, Danuta Barnat-Hunek, Dariusz Majerek

**Affiliations:** 1 Faculty of Environmental Engineering, Lublin University of Technology, Nadbystrzycka Str. 40B, 20-618 Lublin, Poland; Z.Suchorab@pollub.pl (Z.S.); M.Widomski@pollub.pl (M.K.W.); 2 Faculty of Civil Engineering and Architecture, Lublin University of Technology, Nadbystrzycka Str. 40, 20-618 Lublin, Poland; D.Barnat-Hunek@pollub.pl; 3 Faculty of Fundamentals of Technology, Lublin University of Technology, Nadbystrzycka Str. 38, 20-618 Lublin, Poland; D.Majerek@pollub.pl

**Keywords:** time domain reflectometry, TDR, frequency domain, FD, porous materials, building materials, moisture

## Abstract

The article presents the potential application of the time domain reflectometry (TDR) technique to measure moisture transport in unsaturated porous materials. The research of the capillary uptake phenomenon in a sample of autoclaved aerated concrete (AAC) was conducted using a TDR sensor with the modified construction for non-invasive testing. In the paper the basic principles of the TDR method as a technique applied in metrology, and its potential for measurement of moisture in porous materials, including soils and porous building materials are presented. The second part of the article presents the experiment of capillary rise process in the AAC sample. Application of the custom sensor required its individual calibration, thus a unique model of regression between the readouts of apparent permittivity of the tested material and its moisture was developed. During the experiment moisture content was monitored in the sample exposed to water influence. Monitoring was conducted using the modified TDR sensor. The process was additionally measured using the standard frequency domain (FD) capacitive sensor in order to compare the readouts with traditional techniques of moisture detection. The uncertainty for testing AAC moisture, was expressed as RMSE (0.013 cm^3^/cm^3^) and expanded uncertainty (0.01–0.02 cm^3^/cm^3^ depending on moisture) was established along with calibration of the applied sensor. The obtained values are comparable to, or even better than, the features of the traditional invasive sensors utilizing universal calibration models. Both, the TDR and capacitive (FD) sensor enabled monitoring of capillary uptake phenomenon progress. It was noticed that at the end of the experiment the TDR readouts were 4.4% underestimated and the FD readouts were overestimated for 12.6% comparing to the reference gravimetric evaluation.

## 1. Introduction

Among the numerous available methods for estimation of porous media water content, the time domain reflectometry (TDR) technique is considered as one of the most useful [1,2]. Contrary to the capacitive methods, including the frequency domain (FD) or resistance-based methods of moisture measurements, TDR allows moisture determination with satisfactory accuracy, regardless of external factors, including e.g., temperature and, to a certain extent, salinity, affecting the obtained results [3,4,5].

The first historical applications of TDR method in soil water content measurements were reported in the 1980s [6]. Thereafter, the method has been constantly developed and refined, using ongoing achievements in the field of electronics and sensor construction [7,8,9,10,11,12,13,14,15] as well as the required calibration procedures [6,16,17]. A notable increase in the range of possible applications of TDR method also became apparent [18,19,20].

The TDR method generally utilizes observations of the electromagnetic pulse propagation time along the sensor placed in the material that moisture is being investigated. The dimensionless apparent permittivity *ε*, being a measure of molecules’ behaviour under the alternating electromagnetic field and energy dissipation of the material after electromagnetic field is released, is a basic, fundamental parameter required for successful TDR application [6,21,22,23,24,25,26,27,28,29,30,31,32,33].

Several factors affect the apparent density values of multiphase porous media, including their structure, particle size distribution, etc. but the dipolar character of water molecule makes the influence of water the most important. The electric load distribution for water, resulting in the high value of relative apparent permittivity reaching 80 [-], is different than for the other phases of the porous media [21]. The reported values of dimensionless apparent permittivity for air, granite, sandstone, clay and sand were equal to 1, 4−9, 2−3, 2−6, 4−5, respectively [21].

The dielectric permittivity of the materials is a complex number, consisting of a real (ε′) and an imaginary (ε″) part. The real part describes the base value for moisture estimation using the TDR technique, i.e., the amount of released energy in the alternating field, while the imaginary part covers energy loses due to the ionic conductivity, highly dependent to salinity of the medium [23]. The complex dielectric permittivity of saline medium may be calculated according to the following formula [4,22]:

(1) $$\varepsilon_{\omega }=\varepsilon_{\omega }^{\prime }-i\left(\varepsilon_{\omega }^{\prime \prime }+\frac{\sigma_{0}}{\varepsilon_{0}\omega }\right)$$

where: *ε′_ω_*—real part of dielectric permittivity of medium at *ω* frequency [-], *ε″_ω_*—imaginary part of dielectric permittivity of medium at *ω* frequency [-], *i*—imaginary unit (*i*^2^ = −1), *σ_0_*—electrical conductivity [S/m], *ε*_0_—dielectric permittivity of vacuum (*ε*_0_ = 8.85 × 10^−12^ F/m), *ω*—angular frequency of the external electric field [1/s].

The above formula explains that the imaginary part influences measurements in low frequencies of electromagnetic field, e.g., applied in the FD method. The operating frequency of many of the TDR multimeters reaches values of approx. 1 GHz [4], high enough to minimize the influence of imaginary part on the value of complex dielectric permittivity of a saline medium. Thus, it can be assumed that the ionic conductivity has low effect on the TDR readouts, which may be stated as one of the most important advantages of this method in relation to the others based on resistance and capacitance. It must be underlined here, that salinity influences the responses of the reflectometric traces—amplitude of the pulse diminishes and the measuring peaks are flatten, which in some cases may result in the decrease in information available from the tested medium affecting the measuring accuracy [4]. On the other hand, it should be also mentioned, that amplitude diminishing, recognized as a negative phenomenon in the TDR measurement, can be also utilized to evaluate medium salinity of the medium, basing on suitable, insightful waveform interpretation [24].

Therefore, the following formula may be used to calculate the relative apparent permittivity of the porous material [9]:

(2) $$\varepsilon ={\left(\frac{c\cdot t_{p}}{2L}\right)}^{2}$$

where: *c*—light velocity in vacuum [m/s], *t_p_*—travel time along the TDR sensor [s], *L*—length of measuring elements of the TDR sensors [m].

**Figure 1:**
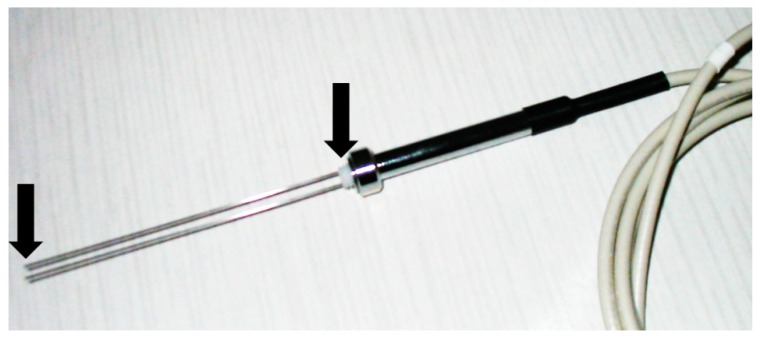
LP/ms probe (ETest, Lublin, Poland) for moisture determination using TDR method.

Moisture measurement using the TDR method rely on the determination of the electromagnetic pulse travel time along the rods of the TDR probes (Figure 1), which generally consist of a concentric cable, head and measuring rods buried into the tested material. The readouts are based on the reflections on particular discontinuities of the sensor waveguide, being the elements of its construction. Usually, the described discontinuities are located at the beginning and the end of probe. Figure 1 shows an exemplary TDR probe, with black arrows marking the discontinuities of waveguide for the electromagnetic pulse. During measurement the rods have to be inserted into the tested material. The contact between the rods and tested material should be precise and permanent to allow the reliable readouts. 

**Figure 2:**
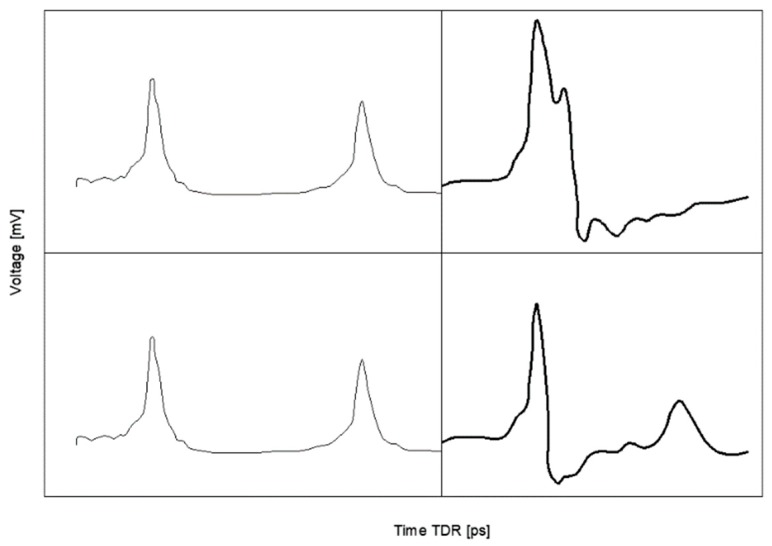
Example of TDR waveforms for dry (**top**) and moist (**bottom**) material acquired from an ETest LP/ms TDR probe (own elaboration based on calibration tests). Left-hand side—control peaks, right-hand side—measuring peaks.

The TDR technology utilizes two types of pulses emitted by the pulse generators: the step pulse and the needle pulse. Both differ in the length of the incident pulse, in the first case the emitted pulse is wider in comparison to the needle pulse length. The exemplary waveforms obtained by the TDR multimeter utilizing 300 ps rise-time needle pulse generator [11] are presented in Figure 2.

They represent the responses of TDR probe on dry and wet material tested by the TDR LP/ms probe (ETest, Lublin, Poland) where the upper trace is representative for the dry materials with low value of the apparent permittivity and the bottom trace represents wet material. The visible and marked differences between peaks of the TDR traces for dry and wet material depend on the apparent permittivity values of the tested material. The left-hand side of both waveforms presents testing peaks, which are not influenced by material moisture. The right-hand side peaks are the measuring ones. The first, positive measuring peak is constant for both, dry and moist, materials. It represents the reflection from the probe input (right black arrow in Figure 1) and the second one, with smaller voltage, representing the reflection from the end of the probe (arrow at the end of the rod). Distance between both measuring peaks expressed in time can be recalculated into the apparent permittivity using Equation (2). The longer time of signal propagation for wet material results in shifting the second measuring peak towards the right side of the graph. Determination of moisture in porous materials based on the measured dielectric permittivity can be accomplished using various theoretical and physical models [25,26,27] or the empirical calibration formulas obtained by the experimental examinations [6,16,17,28].

The significant advantage of the physical models is their independence from calibration procedures. On the other hand, the most essential of their disadvantages is the complicated mathematical description hindering laboratory measurements. The physical descriptions of dielectric parameters of porous materials as ternary mixtures were elaborated in 1892 by Rayleigh [34], in 1904 by Maxwell Garnett [35], and in 1946 by Polder and van Santen [36]. Among the present dielectric models of porous media there should be mentioned the models by De Loor [25], Tinga [26], Roth [37], Whalley [38] and Noborio [28]. All the above mentioned models differ in the approach to the porous material structure, geometry, shape and morphology of the grains but also differ in the grade of complexity and, as it was mentioned above, they are not easy for the practical aspects of moisture evaluation in the real laboratory or in-situ conditions.

The other approach of calibration of the TDR probes for moisture determination is to describe the dielectric parameters of the moist porous media and to develop an empirical model based on laboratory tests allowing the correlation between the gravimetric and TDR moisture readouts. Among the empirical models universal and individual models can be distinguished. The universal models are developed on the base of the multiple investigations of numerous media to describe various materials that differ in density, porosity and structure solid phase. The individual models are elaborated to find calibration formula for the particular material or even sensor.

Among most cited universal empirical models two the most important should be mentioned: Topp’s [6] and Malicki’s [16] formulas. The first is the third order polynomial function relating the moisture of porous material to only one measured parameter – apparent permittivity. This enables quick estimation for many porous materials without the prior calibration independently on the examined material and sensor used. On the other hand, this method not always provides the correct results of the readouts. According to Schapp et al. [39] the possible uncertainty of measurement can vary in the range between 0.05 and 0.15 cm^3^/cm^3^, which may be caused by the differences of solid phase structure of the examined material. According to Černý [11], standard uncertainty of moisture estimation by the Topp’s model equals 0.0468 cm^3^/cm^3^. Additionally, it should be considered that Equation (3) is applicable for porous media with bulk density close to 1500 kg/m^3^, only for volumetric moisture content below 0.5 cm^3^/cm^3^ and should not be used for organic soils or mineral soils containing organic material and clay [24,40].

Malicki’s approach improves the accuracy of moisture determination using the TDR technique compared to the Topp’s model and extents its application. It is described by the semi-empirical formula considering bulk density of the tested material, a part of the apparent density.

The semi-empirical models are still universal and present the acceptable accuracy making them common in reflectometric investigations. On the other hand, many individual calibration formulas elaborated for the particular materials or sensors and offering the better accuracy than empirical models by Topp and Malicki may be found in the literature [19,41,42,43,44,45,46].

## 2. Concept of the Surface TDR Sensor

The building materials present the large share among the porous media. In moderate climate the housing sector suffers from deteriorating water presence inside the building envelopes. Water present in porous building materials decreases their bearing as well as the thermal properties, negatively influencing energetic performance of the buildings. Another negative effect of water presence in the building envelops is the risk of microbial threat and Sick Building Syndrome (SBS) symptoms.

Construction of the previously described traditional TDR probes significantly constricts moisture measurements in the firm porous media, including most of the building materials. The above is triggered by the geometrical and mechanical properties of measurement units—steel rods. They are usually quite long and thin, and, like in case of the LP/ms probes made by ETest, also frail. Such probes are useful during measurements of soil moisture, but in case of water content determination for hard building materials they are inapplicable.

Thus, most of the reported studies concerning water content of building materials were performed under the laboratory conditions allowing the proper preparation of samples. The preparatory activities usually covered drilling the pilot holes in which the rods of the probe were inserted or drilled holes of larger diameter in which void air space was filled with the drilling dust [47,48,49,50]. Unfortunately, all these procedures were altering the structure of studied material, including its water characteristics. Thus, the obtained readouts for the transformed material were not reflecting the real moisture conditions of the studied sample. There are two possible concepts of solving the problem of moisture measurements in firm building materials:

- construction of TDR probes of significant size, consisting of steel rods of the required diameter and durable head [4];
- construction of the TDR surface sensor.

The first surface TDR sensor concepts were reported in the 1990s [14,15]. The probe proposed by Selker et al. [14] utilized the long brass wire, shaped in the spiral manner, covered by acrylic plate. The individual *ε*-*θ* calibration was required for this probe and its measurements uncertainty wearied in the range ±0.02 cm^3^/cm^3^. On the other hand, the idea of sensors proposed by Perrson and Berndtsson [15] was based on application of the typical three-rod probes covered by the properly carved dielectric of known thickness and dielectric characteristics, allowing determination of dielectric parameters, thus water content, of medium located below the cover. This solution was rather primitive but it enabled, to some extent, the non-invasive determination of moisture in porous medium.

The interesting and different solution of surface probe was proposed by Wraith et al. [51] as the probe for determination of moisture in top soil. The probe similar to sledges could be pulled over the soil surface like the georadar, allowing measurements of top soil water content.

Ito et al. [52] proposed the multi-TDR probe, allowing measurements of evaporation from soil surface, consisting of the layered composite of glass and resign, covering 17 copper electrodes in shape of stripes, 100 mm length, 0.02 mm width and 0.01 mm thick. The unit was consisting of 8 combined probes, for which the individual calibration was required. 

The new concept of non-invasive TDR sensors was proposed by Choi et al. [53]. The three-rod surface probe was additionally equipped in the piezoelectric sensor and accelerometer, allowing the measurements of dry bulk density, soil moisture and modulus of elasticity. All the measurements may be performed without altering the soil surface.

The concept of the TDR surface sensor for firm materials was presented in the patent reservation [54]. The prototype and possible applications were already reported [4]. Modifications of this TDR sensor allowing moisture measurements in firm porous materials of irregular surface were also presented in patent’s documentation [55,56,57].

The performed literature studies showed that time domain reflectometry is a very applicable technique in the field of soils science. However, it was also indicated that there is a need for development of the method allowing TDR application in determination of wall barriers moisture conditions. Thus, construction of the probes and development of the required measurement methodology are required.

The aim of studies presented in this paper was to apply the indirect moisture detection technique, TDR with the modified sensor construction, to determinate the unsaturated water flow in a rigid, porous building material. The conducted research was additionally supplemented with the FD (capacitive, non-invasive probe and direct gravimetric evaluation). The aerated autoclaved concrete (AAC) was selected as a tested material, due to popularity of the material in modern building sector but also the proper hygric parameters that would reveal the measuring potential of the tested sensors: low density, high porosity, high capillarity, saturation, etc.

## 3. Materials and Methods

### 3.1. Details of the Developed Sensor

**Figure 3:**
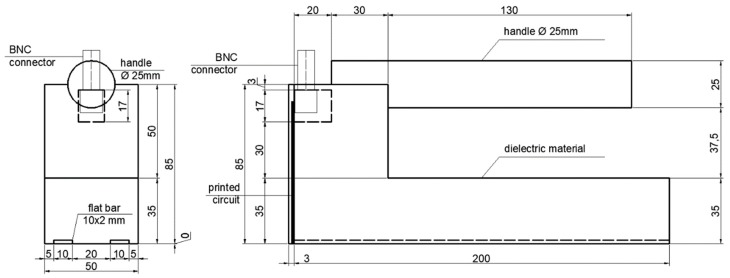
Schematic view of the proposed sensor construction.

The subject of study was a TDR surface sensor developed to examine moisture content of rigid porous media as building materials, building barriers or rocks. The prototype specimen applied for the presented research was constructed of black polyoxymethylene (POM)—plastic characterized by good mechanical parameters including strength, stiffness, ductility and value of apparent permeability at the level of 3.8 [7]. The length of the probe and waveguide was equal 200 mm, while width 50 mm. Measuring elements were manufactured from brass flat bar 2 mm × 10 mm. The device was equipped with a cylinder shaped handle. Communication between the probe and the TDR multimeter was provided by the BNC connector with simple printed circuit consisting of the two lines soldered to the pins of the BNC connector and both bars on the other side. There was a resistor soldered between two lines. A schematic view of the proposed surface TDR sensor construction is presented in Figure 3.

**Figure 4:**
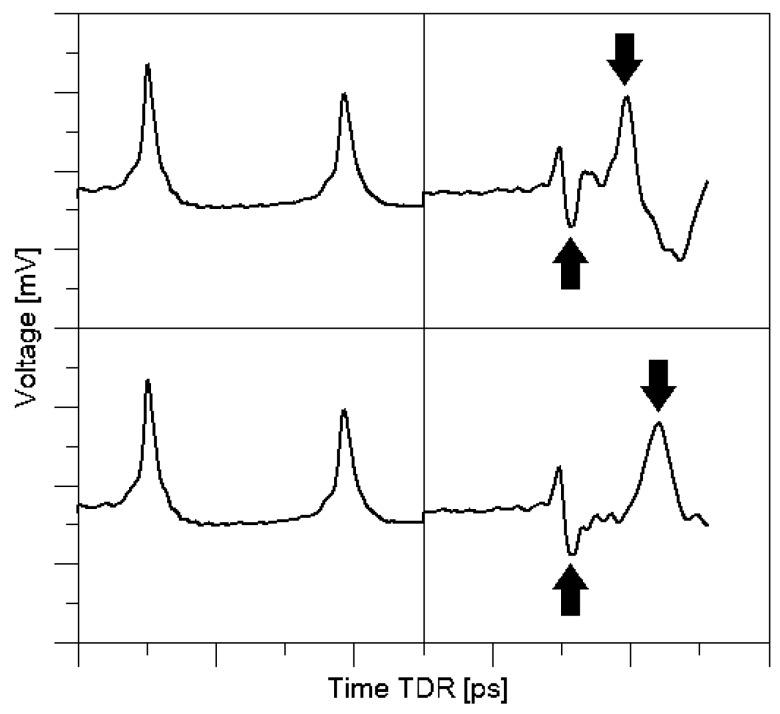
Electric response of the developed sensor for different environments: upper trace—air-dry sample, bottom trace—saturated sample.

Figure 4 presents the TDR traces obtained with the discussed sensor. 

The first negative peak (marked with an arrow) is constant in position and is a consequence of mounting of the resistor in the printed circuit of the sensor. The second, positive peak (marked with arrow) means the measuring element termination and its position results from material moisture.

Before the sensor was used for laboratory experiments it was tested to define the range of electromagnetic signal influence in the measured material. From the literature it is known that this mainly depends on the spacing between the two measuring waveguides [58]. This parameter of the developed sensor was evaluated in laboratory conditions, methodology of its estimation was described in the following article [59]. In case of the described sensor the range of signal influence was defined as 40 mm deep.

### 3.2. Measuring Setup

The following materials were applied to the experiment:

- Aerated concrete, dry apparent density 600 kg/m^3^;
- Laboratory oven VO-500 (Memmert, Schwabach, Germany);
- Bitumen isolation;
- Laboratory scale WPT 6C/1 (RADWAG, Radom, Poland);
- Multifunctional scale WPW 30/H3/K (RADWAG, Radom, Poland),
- Water reservoir equipped with necessary equipment to sustain the constant water level;
- TDR equipment including laboratory multimeter LOM (ETest, Lublin, Poland);
- TDR sensor presented in this article, concentric cable;
- Personal Computer for meter control and data management;
- Capacitive moisture meter LB-796, (LABEL, Reguły, Poland);
- Atomizer (for calibration procedure).

### 3.3. Preliminary Research

Preliminary research was conducted to establish the basic physical and hygric parameters of the materials, important from the point of view of the conducted experiment: apparent density of the material and its saturated water content. Three samples 50 mm × 50 mm × 45 mm were prepared and dried in the 105 °C in the laboratory oven. After dry mass was determined, the samples were saturated to allow determination of gravimetric and volumetric water contents. Gravimetric and volumetric water content were determined using the following equation [29]:

(3) $$w=\frac{m_{n}-m_{s}}{m_{s}}$$

(4) $$\theta_{V}=\frac{V_{w}}{V_{tot}}$$

where: *w*—gravimetric water content [kg/kg], *m_n_*—mass of wet sample [kg], *m_s_*—mass of dry sample [kg], *θ_V_*—volumetric water content [cm^3^/cm^3^], *V_W_*—volume of water [cm^3^], *V_tot_*—total volume of the sample [cm^3^].

### 3.4. Calibration the Sensor

Unusual sensor construction and insufficient verification of calibration formulas, intentionally developed for soils, caused the necessity of the individual calibration procedure for the newly developed sensor which was going to be applied for building materials.

The dimensions of the samples was the following: 220 mm × 120 mm × 40 mm. External surfaces of the samples were polished to provide equal adherence to the tested material. The first step was conducted on the dry set of the samples. Then the samples were sequentially moistened using atomizer with steady portions of water to achieve the full saturation. During the experiment the samples were weighed using laboratory scale and volumetric water content was evaluated using the Equation (4). Then the surface sensor was pressed to the tested sample with constant pressure and the effective dielectric permittivity was read. For the statistical post-processing, each step of measurement was repeated five times.

### 3.5. Model of Regression

According to the authors’ experience and the literature [41,42] the assumed general form of calibration equations (see Equation (5)) has a second order polynomial function character. The input data for the model covered volumetric water content obtained due to the direct gravimetric measurements and the mean value of the effective dielectric permittivity obtained by the reflectometric measurements:

(5) $$\begin{array}{l} \theta =\beta_{0}+\beta_{1}\cdot \bar{\varepsilon_{eff}}+\beta_{2}\cdot {\bar{\varepsilon_{eff}}}^{2}+\epsilon \end{array}$$

(p)      (p)      (p)

where: *θ*—volumetric water content determined by polynominal model [cm^3^/cm^3^]; $\bar{\varepsilon_{eff}}$—mean effective dielectric permittivity obtained by reflectometric measurements [-], ϵ—random error of normal distribution, *p*—critical level of significance (* *p* < 0.05; ** *p* < 0.01; *** *p* < 0.001).

### 3.6. Calculation of Uncertainty

The measurements uncertainties type A determine the quality of models’ fitting to the experimental data. The source of type B of uncertainties are the measuring uncertainties of the instruments used within the calibration procedure. In the Equation (5) there are two sources of variance. The first is the regression uncertainty and *ε~N*(0, *σ*) which comes from the randomization which results in some variability of all estimated regression parameters, that is expressed in covariance matrix *σ*^2^*(X’X)*^−1^. The second source is a consequence of fact, that it is not possible to reveal the true dependence between the selected predictors and the dependent variable—fitted volumetric water content value may differ from the true value of *θ* because it is impossible to control all variables affecting it [60]. Uncertainties type B are neglected from the investigation because they are of lower level comparing to the uncertainty of A type. In the assumed model four factors affect measurement uncertainty: estimators *β*_0_, *β*_1_, *β*_2_, and the dielectric permittivity:

(6) $$\theta =f\left(\beta_{0},\beta_{1},\beta_{2},\varepsilon \right)$$

Using the error propagation law, the combined standard measurement uncertainty (including uncertainties type A and B) may be presented as follows [60,61]:

(7) $$u_{C}\left(\theta \right)=\sqrt{{\left(\frac{\partial \theta }{\partial \varepsilon }u\left(\varepsilon \right)\right)}^{2}+\overset{2}{\underset{i=0}{\sum }}{\left(\frac{\partial \theta }{\partial \beta_{i}}u\left(\beta_{i}\right)\right)}^{2}+2\overset{2}{\underset{i=0}{\sum }}\overset{2}{\underset{j=i+1}{\sum }}\frac{\partial \theta }{\partial \beta_{i}}\frac{\partial \theta }{\partial \beta_{j}}u\left(\beta_{i},\beta_{j}\right)}$$

so:

(8) $$u_{c}{}^{2}\left(\theta \right)=S^{2}\left(1+\frac{1}{n}+\sum_{i}{\left(\frac{\partial \theta }{\partial \beta_{i}}\right)}^{2}u^{2}\left(\beta_{i}\right)+2\sum_{ij}\left(\frac{\partial \theta }{\partial \beta_{i}}\frac{\partial \theta }{\partial \beta_{j}}\right)cov\left(\beta_{i}\beta_{j}\right)\right)$$

The expanded measurement uncertainty was determined using the following formula:

(9) $$U\left(\theta \right)=k_{p}\cdot u_{c}\left(\theta \right)$$

where: *k_p_*—coverage factor, calculated from t-student distribution for *α* = 0.05, depending on the number of degrees of freedom, it oscillates around 2.

### 3.7. Capillary Suction Test

**Figure 5:**
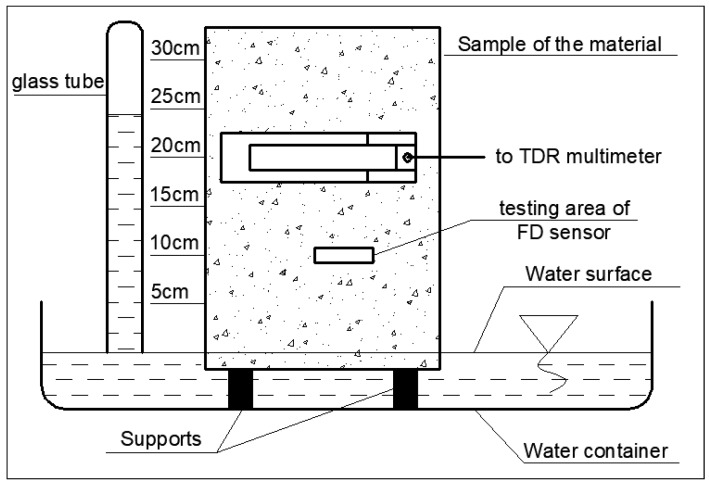
Schematic view of the capillary uptake setup.

The aim of the laboratory research was to assess the measurement potential of the prototype TDR sensor and to demonstrate its applicability in practical aspects as well as to compare its measuring features with a popular moisture sensor available on the market. The research was focused on monitoring of water transport in the model aerated concrete wall barrier utilizing newly developed TDR sensor and the Label LB-796 capacitive FD sensor (LABEL, Reguły, Poland), providing satisfactory readouts of moisture in building barriers, being successfully used for expertises concerning water damage of the buildings. The applied sample was cut from the concrete block to the dimensions of 240 mm × 240 mm × 350 mm, dried to constant mass and covered with thin layer of the bitumen isolation in order to minimize the environmental impacts on the studied process. The scheme of capillary rise monitoring in aerated concrete is presented in Figure 5. The studied sample was inserted approx. 1 cm below the distilled water surface in the reservoir. The water level was kept constant with the help of the glass tube filled with water. The measurements points were assigned in 5 cm interval (levels 5, 10, 15, 20, 25 and 30 cm) above the water surface. During the measurement the TDR sensor was carefully contacted to the tested sample maintaining the constant pressure. The middle of its width was positioned at the particular measuring levels as visible in Figure 5. The similar procedure was repeated for the applied FD sensor, according to the producer’s recommendations.

Before and after the capillary suction test the sample was weighed, which enabled verification of the indirect readouts (by the tested surface TDR and capacitive sensors) to the direct readouts obtained gravimetrically. The mass of sample dried in temperature of 105 °C was equal to 12.39 kg while the determined apparent density reached the value of 614.6 kg/m^3^ and was higher than declared by the producer. The determined mass of the moist concrete sample after capillary rise test was equal to 16.91 kg, thus the increase of 4.52 kg was observed in relation to mass before the test.

The TDR and LB-769 studies were performed for a time duration of 16 days. Moisture readouts were performed three times a day. Each measurement was based on contact of the TDR and capacitive sensors with the studied sample at the given height. During the measurement a constant pressure between both sensors and the tested material was kept to minimize the influence of uneven contact condition on measuring accuracy. Each measurement was repeated three times, in order to assure the statistically required number of data. The environmental conditions of measurements were as follows: temperature 20 °C ± 2 °C, relative air moisture 50% ± 5%. The curves presenting the dynamics of capillary rise process by the sample of tested aerated concrete were obtained as the result of the experiment. The values of effective permittivity *ε_eff_* were converted into the volumetric moisture content, using the calibration formula obtained within the calibration test (Section 3.4 and Section 3.5). The FD sensor readouts were performed in the similar manner, simultaneously to the TDR measurements, according to the producer’s guidelines. The FD meter was pre-calibrated by the producer, which enabled reading ready values of moisture content. Since the FD results are presented as the gravimetric water content, the readouts were converted to volumetric water content.

## 4. Results

### 4.1. Preliminary Test Results

The basic hygric parameters of the materials were established with the preliminary tests. Apparent density, volumetric and gravimetric saturated water content of the studied material are presented in the Table 1.

### 4.2. Calibration of the TDR Sensor

**Figure 6:**
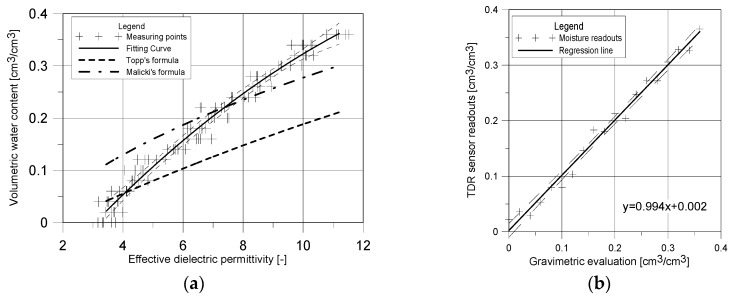
Calibration test results: (**a**) dependence between effective dielectric permittivity and material moisture, (**b**) comparison of data obtained gravimetrically and by reflectometric evaluation.

With the calibration procedure the dependence between effective dielectric permittivity (read by the TDR surface sensor) and the volumetric water content was achieved. The results are presented in Figure 6a.

As it was previously mentioned, the dependence between data presented in Figure 6a can be described using the second order polynomial regression model proposed as Equation (10):

(10) $$\hat{\theta }=-0.1956\;+\;0.0691\varepsilon_{app}-0.0017\;\varepsilon_{app}^{2}$$

(***)      (***)             (*)

The basic statistical parameters of the developed regression formula are stated in Table 2.

The comparison of results obtained by model for the surface TDR probe and the gravimetric measurements were presented as graph in Figure 6b. 

### 4.3. Combined Standard and Expanded Measurement Uncertainty

**Figure 7:**
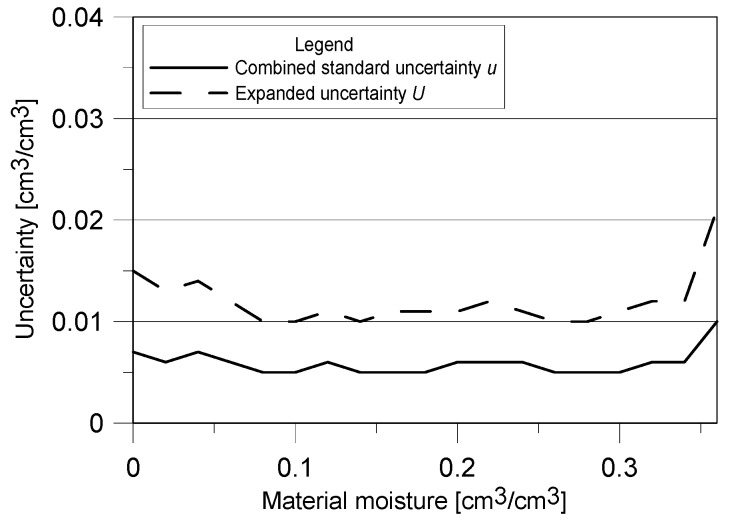
Combined standard and expanded measurements uncertainties of the TDR surface sensor for aerated concrete.

The results of determined (according to Equations (8) and (9)) combined standard and expanded measurements uncertainties were presented in Figure 7.

For most of the material moisture range the expanded uncertainty of TDR measurement using the surface sensor is about 0.01 cm^3^/cm^3^. Only in nearly dry and saturated conditions its value is higher, 0.015 and 0.02 cm^3^/cm^3^, respectively.

### 4.4. Capilary Suction Results

**Figure 8:**
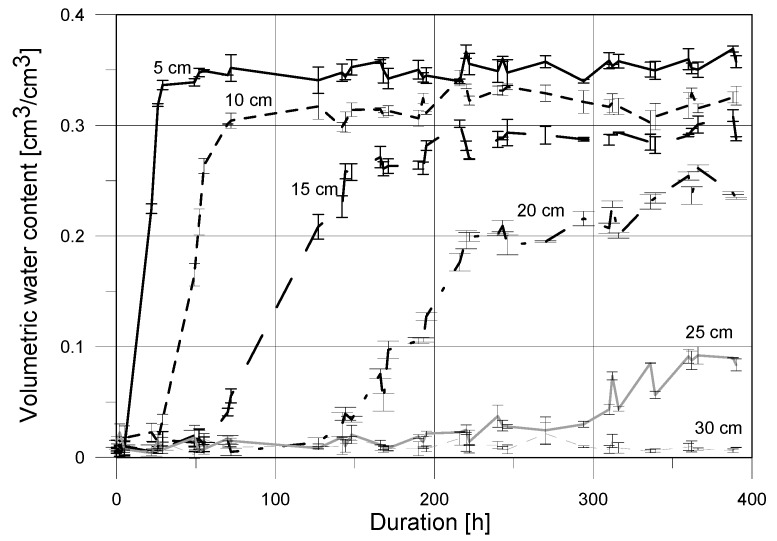
Capillary rise determined by TDR surface sensor in sample of aerated concrete.

The graph presented in Figure 8 shows the curves of capillary rise determined by the applied surface TDR sensor in the reference points at given heights above the water level. It represents the mean values of three repetitions, supported by the standard deviations expressed as error bars. 

**Figure 9:**
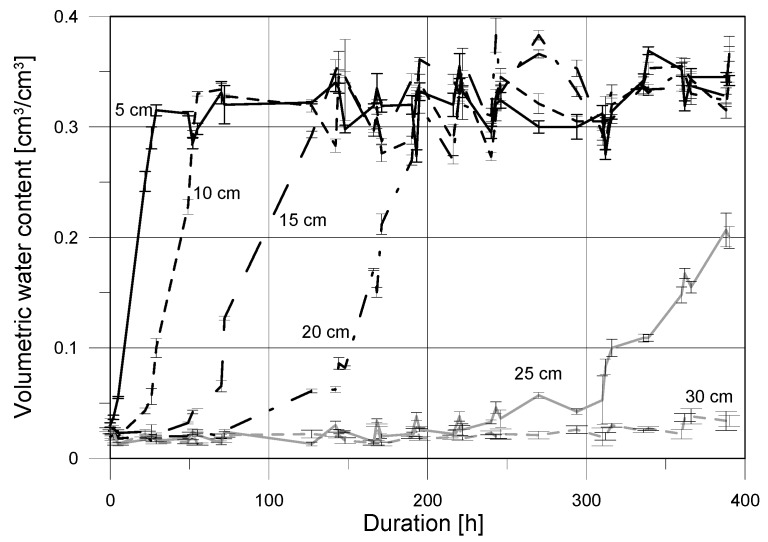
Capillary rise determined by FD capacitive sensor in sample of aerated concrete.

The mean values obtained by the applied FD sensor and supported by SDs values are presented in Figure 9.

The changes of moisture in subsequent reference points determined by the indirect electric measurements were observed. The initial water content presented in Figure 8 and Figure 9 showed values close to zero, and was equal 0.01 cm^3^/cm^3^ and 0.02 cm^3^/cm^3^ when determined by the TDR and FD sensors, respectively. The reported initial readouts slightly greater than zero may be caused by the manner of sample preparation (drying in 105 °C to constant mass) and measurement uncertainty of applied sensors for the assumed model of regression.

The readouts of both applied sensors showed a very fast increase in moisture at the 5 cm reference point. The trend of the increase is clear, which underlines the strong capillary properties of the tested medium. At the beginning of the second day of the experiment the full saturation of medium by water was observed in point located at the height of 5 cm. The increase in water content at higher level was also rather rapid but was shifted in time—at the height of 10 cm the presence of water was noted on the another day of experiment. The discussed increase was also dynamic and within the next day the full saturation conditions were achieved. Increase in water content at the height of 15 cm was less dynamic, comparing to the lower heights. The first presence of capillary water was noted after three days of the experiment, while the full saturation was observed four days later. The fourth reference point, at the height of 20 cm above the water level, showed increase in water content after six days of experiment. Then, the slow gain of moisture was observed, leading to conditions close to full saturation after the next four days. At the height of 25 cm, both sensors (surface TDR and FD), showed increase in water content after 300 h, i.e., after over 12 days, but the differences in reported values, reaching 0.1 cm^3^/cm^3^ for the TDR sensor and 0.2 cm^3^/cm^3^ for FD one, are visible. No increase in water content was observed by both of the probes in the reference point at the height of 30 cm.

## 5. Discussion

### 5.1. Discussion on the Calibration Results and Uncertainty Calculations

According to the assumed model of regression, the second order polynomial formulas were obtained. In order to underline the differences between values of dielectric permittivity obtained by the measurements using the developed TDR sensor and the application of typical invasive probes, there were presented values of water content for the respective values of the dielectric permittivity obtained by formulas by Topp [6] and Malicki [16].

In case of dry samples and moisture contents below 0.05 cm^3^/cm^3^ the effective apparent permittivity determined using the surface TDR sensor reaches the values in the range between 3 and 4. This is the consequence of the values of apparent permittivity of solid phase of material and apparent permittivity of polyoxymethylene that equals 3.8. In the higher ranges of moisture the readouts of apparent permittivity by the TDR surface sensor show the greater moisture than values read by the traditional invasive probe using Topp’s or Malicki’s calibration formulas. This is mainly caused by the influence of the polyoxymethylene covering the waveguides and significantly decreases the effective apparent permittivity read by the surface sensor at the particular level of the sample. The estimated calibration Equation (12) considers this influence and precisely reproduces the dependence between the examined moisture and readouts of apparent permittivity by the surface TDR sensor. This is also confirmed by the statistical characteristics of the applied model, mainly coefficient of determination which equals 0.986 and Residual Standard Error (RSE) = 0.014 cm^3^/cm^3^. Also, the linear formula of regression presented in Figure 6b has the following features: slope value equal 0.994 and y-intercept value equal 0.002. Levels of significance of particular parameter estimators in the Equation (10) are lower than 0.001 in case of *β*_0_ and *β*_1_. Only in the case of estimator *β*_2_ the significance level is below 0.05. Simultaneously, the analysis of F Statistic (*p* < 0.001) confirms the statistical significance of the applied model. Root mean square error (RMSE), the frequently used measure of uncertainty, equals 0.013 cm^3^/cm^3^ and is lower that could be found in the literature concerning even the invasive probes. According to the data presented by Ju et al. [46] using the Topp’s model in relation to the selected soils caused uncertainties expressed as RMSE in the range of 0.01–0.066 cm^3^/cm^3^. The RMSE value for the model proposed by Roth et al. [37] was in the range of 0.008–0.037 cm^3^/cm^3^ depending on material, while the RMSE for moisture estimation using the Malicki’s model [16] equals 0.03 cm^3^/cm^3^. The RMSE value obtained for the described surface TDR sensor is smaller than presented in the cited literature, anyway it must be remembered that discussed formulas are universal and because of that the quality of data fitting may be worse. The model presented in this article is individual, dedicated to the particular sensor and material, which may explain the better projection of the discussed dependence *ε_eff_*-*θ*. Analyzing the RMSE value established for the presented sensor it should be mentioned that it is located in the range of RMSE values established by Udawatta et al. [42] for individual models estimated for traditional invasive probes and different materials (0.008–0.034 cm^3^/cm^3^).

Concerning uncertainty determination it must be mentioned, that like RMSE, the obtained values of uncertainties are lower comparing to the traditional invasive sensors calibrated with the standard empirical formulas. As it was mentioned in Section 4.3, lower and higher ranges of moisture are characterized with the greatest value of measuring uncertainty and the lowest values are noted in the middle range of moisture values available for the tested material. This is the feature of most measuring devices, in this particular case it is caused by the applied model of regression [62]. According to Topp et al. [63] and Amato and Ritchie [64] the uncertainty of measurement ranges between 0.022 and 0.023 cm^3^/cm^3^, according to Černý [11]—0.0269 cm^3^/cm^3^, Malicki et al. [16] 0.004–0.018 cm^3^/cm^3^ and finally Roth et al. [37] 0.011–0.013 cm^3^/cm^3^. The expanded uncertainty obtained for the presented noninvasive sensor and the tested material is within the values declared by the cited authors for the traditional invasive sensors or even lower. In the opinion of the authors of this elaboration, the beneficial measuring parameters of the prototype non-invasive TDR sensor are the consequence of the following reasons:

- model of regression is individual;
- most of the cited models were developed for soil media, less homogenous in comparison to the tested building material (autoclaved aerated concrete).

### 5.2. Discussion on Capillary Uptake Experiment Results

**Figure 10:**
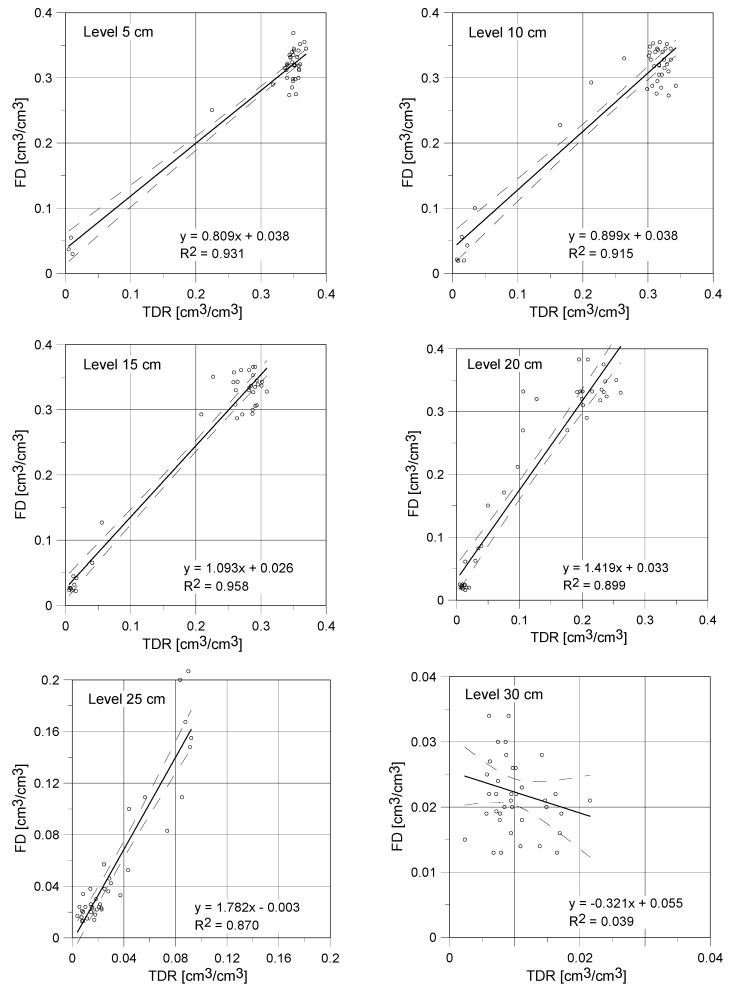
Comparison of capillary rise measurements results obtained by surface TDR sensor and FD capacitive sensor for aerated autoclaved concrete.

Progress of capillary uptake examined using the two applied indirect techniques was presented in Figure 8 and Figure 9 and commented in Section 4.4. Figure 10 shows the comparison of moisture readouts in the individual reference points obtained by the surface TDR and FD sensors. It is visible, that the presented curves are similar for the measurements performed close to the water table level.

For the reference levels at 5, 10 and 15 cm the obtained slopes of linear regression equation were equal to 0.809, 0.899 and 1.093, respectively. Thus, for the first two points the applied TDR noninvasive sensor reports higher values of moisture. Contrary, in case of the third point (15 cm), higher values were shown by the FD capacitive sensor. It should be also noticed that y-intercepts values are slightly above zero in most circumstances, which means that the FD sensor shows higher moisture values under dry conditions. Since this level, the differences between both readouts are more visible. At the height of 20 cm the obtained directional coefficient was equal to 1.419. So, the FD sensor reported water content significantly higher than the TDR. This tendency was noted also for the reference points located in higher elevations, the directional coefficient for linear regression was equal to 1.782 at the level of 25 cm. The huge differences between the TDR and FD sensors were also observed for the reference point at the level of 30 cm (the negative value of regression coefficient). 

These differences are probably related to low values of the compared readouts (both sensors reported values from range 0–0.04 cm^3^/cm^3^) and high measurement uncertainty for the low range of determined water content. All observed differences between both techniques of moisture detection are the consequence of their indirect character and the potential influence of some disturbances which not always could be minimized or eliminated, for example ionic conductivity, contact condition and nonhomogeneous degree of material saturation.

The laboratory studies performed with the application of two types of sensor showed the close moisture readouts and the similar trends of water content changes, both, in its dynamics and quantitative aspect. The following differences were observed:

- moisture readouts at points located at low height about the water table (5 and 10 cm) were higher for the TDR noninvasive sensor;
- at the height of 15 cm moisture content determined by capacitive probe was slightly higher that one indicated by the TDR sensor which is confirmed by the slope of regression higher than 1 and positive value of the y-intercept;
- for low saturation conditions the FD probe showed higher moisture readouts than the TDR surface sensor;
- both of the tested probes showed high measurement instability for low saturation (close to dry), which is visible in Figure 10 for the reference level at 30 cm, with the negative coefficient of regression;
- the maximal noted standard deviation for the TDR sensor was equal to 0.012 cm^3^/cm^3^ with the maximal standard deviation for the FD probe was higher, reaching 0.037 cm^3^/cm^3^.

The comparison of sample’s mass before and after experiment was performed by the standard procedure and showed the difference of 4.52 kg. In case of the applied electric methods the amount of absorbed water was determined by integration of moisture profile observed at the end of the experiment. The following formula was applied:

(11) $$m=0.001\cdot a\cdot b\overset{h}{\underset{0}{\int }}\theta \left(h\right)dh$$

where: *m*—mass of water absorber by the tested material [kg]; *a*, *b*—dimensions of sample: width and depth (24 cm); *h*—height of the sample; *θ(h)*—water profile for the final time duration of the experiment.

It was determined that for the final part of the experiment the increase in water mass determined by the TDR surface sensor was equal to 4.32 kg and 5.09 kg for the capacitive probe. Thus, the increase in water mass estimated with application of the TDR and FD probes was 4.4% lower and 12.6% greater, respectively, than the increase obtained by the gravimetric method. Calculated underestimation of the increase in water mass by the TDR surface sensor may be related to its range of signal influence, equal to 4 cm, while the thickness of the sample was equal to 24 cm. Assuming the heterogeneous structure of tested material and complex process of water transport, it may be accepted that some part of water was unavailable for the TDR and FD sensors impulse. On the other hand, moisture overestimation presented by the FD probe may be influenced by salt ions present in water inside the tested porous material.

Due to the unique prototypes of probes, different physical characteristics of tested material and its heterogeneity, it is hard to relate the obtained results to the literature reports. The TDR technique is being actually introduced to measurements of water content in rigid porous building materials, so a few literature reports allowing comparison of the results are available. Moreover, the reported results concerning moisture changes in samples of building materials were obtained by the invasive or direct methods.

The measurements of capillary rise in the sample of aerated concrete utilizing the invasive TDR probes were performed by Hansen [47] and during the earlier studies by Suchorab et al. [48]. The aerated concrete researched by Hansen [47] had apparent density of 500 kg/m^3^, lower than tested in this paper, so installation of the invasive TDR probes could be easier. The probes were installed at the following heights over the water table 15, 30, 45, 60, 75 and 90 mm, lower and with smaller spacing than applied in our research. Thus, the first registered readouts of water content for the lowest level, 5 cm above water level, were observed after approx. an hour and after 5 h the conditions near full saturation were noted. The increase in water content for higher levels were observed respectively later. To compare the dynamics of the studied processes, the readouts of water content at the height of 90 mm reported by Hansen [47] and 100 mm obtained during the presented studies were analyzed. In case of the surface TDR probe the appearance of water was observed after approx. 20 h and the full saturation after approx. 80 h, while the comparable values were reported by Hansen [47] after approx. 60 and 100 h, respectively. But, the full saturation was probably not achieved, because lower sensors showed higher values of moisture readouts in several points. 

The another quoted paper [48] presented results of the similar studies concerning monitoring of capillary rise in aerated concrete by the invasive field ETest FP/mts TDR probes. In this study, the density of applied concrete sample (24 cm × 16 cm × 6 cm) was equal to 500 kg/m^3^. The initial conditions showed volumetric water content at the level of 0.1 m^3^/m^3^. The TDR FP/mts probes were installed at the heights of 5, 10, 15 and 20 cm above the water table, similarly to the experiment concerning application of the FD and surface TDR sensors. The reported experiment lasted 20 days. The maximum value of water content at the end of the experiment was equal to 0.34 cm^3^/cm^3^ and was comparable to readouts by the surface TDR and FD sensors (0.357 and 0.338 cm^3^/cm^3^, respectively). The increase in moisture to full saturation determined by the TDR FP/mts probes appeared at given tested heights after 3, 5, 10 and 20 days, respectively.

In case of the prototype TDR sensor, presented in this article, time duration required for the full saturation for various heights of reference level reached 2, 4, 8, and 12 days. The measurements of rigid porous materials performed by the traditional probes had more stable process and were characterized by lower values of the determined standard deviations, approx. 0.001 cm^3^/cm^3^. Contrary, both, the surface TDR and FD, sensors showed values of standard deviation equal to 0.005 cm^3^/cm^3^, respectively. However, it should be underlined that all the determined values of standard deviations were below the extended uncertainty of TDR method. The observed differences in readouts of porous material water contents were caused by the different physical properties of tested specimens, various characteristics of sensors and varies character of the performed research.

## 6. Conclusions

The research on the prospective application of the surface TDR proved that the time domain reflectometry technique can be successfully utilized for noninvasive determination of moisture of rigid porous materials. Construction of the presented sensor enables to avoid the limitation of the traditional invasive probes, previously utilized only in soil science, and to extend the technology potential to other branches, mainly civil engineering. A thorough analysis of the obtained results enabled formulation of the following conclusions:

(1) For proper recalculation of reflectometric moisture readouts, the noninvasive, surface TDR sensors require individual calibration.
(2) Due to influence of polyoxymethylene cover of the sensor, apparent permittivity read by the noninvasive sensor is lower than one read by the traditional probe in relation to the same moisture level. These differences can be abolished by application of the individual calibration.
(3) Residual mean squared error (RMSE) for the calibration formula developed for the discussed sensor and material equals 0.013 cm^3^/cm^3^ and is smaller than found in the literature for the traditional invasive probes utilizing the standard empirical calibration formulas.
(4) Expanded uncertainty of the discussed sensor equals 0.01 cm^3^/cm^3^ in the most of the range of material moisture which is lower value than found in the literature for the invasive sensors utilizing the traditional empirical calibration formulas.
(5) Expanded uncertainty of the tested sensor is higher at nearly dry and nearly saturated states of the measured material.
(6) In the range of high moisture values, water content readouts by the TDR surface sensor were higher than those acquired by the capacitive sensor.
(7) In the range of average and low moisture values, water content readouts by the TDR surface sensor were lower than those acquired by the capacitive sensor.
(8) During the comparison of the indirect, electric estimation of moisture using noninvasive TDR and FD sensors with the gravimetric evaluation it was noticed that the TDR readouts were underestimated for 4.4% and the FD readouts were overestimated for 12.6%.
(9) Comparing the maximal standard deviations in both tests using electric techniques of moisture detection it was noted, that capacitive sensors are characterized by greater values of this parameter.

## Figures and Tables

**Table 1:** Basic physical properties of the examined material.

Apparent Density [kg/m^3^]	Saturated Volumetric Water Content [cm^3^/cm^3^]	Saturated Gravimetric Water Content [kg/kg]
612.2 ± 11.2	0.363 ± 0.007	0.593 ± 0.007

**Table 2:** Statistical parameters of the developed calibration model of the surface TDR probe.

Determination Coefficient R^2^	Residual Standard Error RSE [cm^3^/cm^3^]	Root Mean Square Error RMSE [cm^3^/cm^3^]	F-Model Linearity Test Statistic
0.986	0.014 (df = 16)	0.013	580.752 *** (df = 2; 18)

*** *p* < 0.001.
